# Supplementary material for: Genetically Engineered Mouse Models of Liver Tumorigenesis Reveal a Wide Histological Spectrum of Neoplastic and Non-Neoplastic Liver Lesions
Source: Cancers (Basel). 2020 Aug 13;12(8):2265. doi: 10.3390/cancers12082265 (PMC7465606; doi:10.3390/cancers12082265)
Supplement: Supplementary file 1 [file cancers-12-02265-s001.pdf]

Supplementary Materials: Genetically Engineered Mouse Models of Liver Tumorigenesis Reveal a Wide Histological Spectrum of Neoplastic and Non-Neoplastic Liver Lesions

Katja Steiger, Nina Gross, Sebastian A. Widholz, Roland Rad, Wilko Weichert and Carolin Mogler

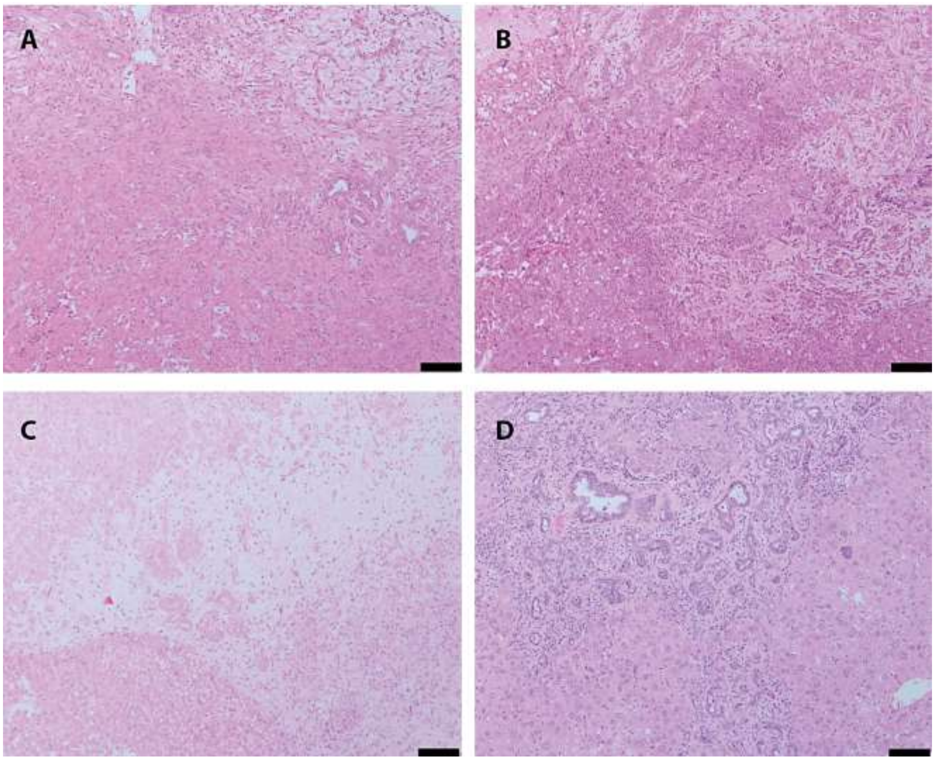

Figure S1. Additional lesions identified in GEMM. (**A,B**) oval cell proliferations; (**C**) inflammation; (**D**) lobular hepatitis. Scale bar: 100 µm.

Table S1. Distribution of age and sex of GEMM

GEMM	Female [%]	Male [%]	Median age [Days]	Range [Days]
PTEN/TGFβR2	55.4	46.6	233	99–730
PTEN	52.3	47.7	346	20–430
PTEN/IDH1	66.67	33.33	607	379–850
KRAS	28.3	71.7	426	236–750
KRAS/PTEN	81.1	18.9	403	305–521

Distribution of age and sex according to specific genetic modification.

© 2020 by the authors. Licensee MDPI, Basel, Switzerland. This article is an open access article distributed under the terms and conditions of the Creative Commons Attribution (CC BY) license (http://creativecommons.org/licenses/by/4.0/).
